# Elevated free triiodothyronine may lead to female sexual dysfunction in Chinese urban women: A hospital-based survey

**DOI:** 10.1038/s41598-017-01352-9

**Published:** 2017-04-27

**Authors:** Han Luo, Hongliu Yang, Wanjun Zhao, Qianqian Han, Li Zeng, Huairong Tang, Jingqiang Zhu

**Affiliations:** 1 Department of Thyroid & Parathyroid Surgery, West China Hospital, Sichuan University, Chengdu, P.R. China; 2 Department of Nephrology, West China Hospital, Sichuan University, Chengdu, P.R. China; 3 Biostatistics Center, West China Hospital, Sichuan University, Chengdu, P.R. China; 4 Health Promotion Center, West China Hospital, Sichuan University, Chengdu, P.R. China

## Abstract

Research on female sexual dysfunction (FSD) is limited, especially in China, due to conservative culture and beliefs. There has been a dearth of FSD screening research in China since the optimal cutoff value of the Chinese version of the Female Sexual Function Index (CVFSFI) was determined in 2014. At the same time, the relationship between thyroid hormones and FSD has seldom been explored in Chinese women. Therefore, hospital-based research was conducted to elucidate FSD frequency and risk factors. Women who underwent a check-up at the Health Promotion Center were approached to participate and, if consented, were enrolled in the study. Demographic and socioeconomic data was extracted. All participants completed the CVFSFI and Beck Depression Inventory (BDI) self-report questionnaires and underwent thyroid hormone tests. A total of 1119 participants were included in the final analysis, with a mean age of 38.6 ± 7.6 years and average CVFSFI score of 25.7 ± 3.9. The frequency of FSD among the participants in this hospital-based cross-sectional study was 26.5%. In addition to age, menopause, parity and depression status as risk factor, and annual income (40,000–100,000 RMB/year) and educational background (≥university) as protective factor, elevated free triiodothyronine (fT3) was identified as an independent risk factor of FSD.

## Introduction

In the 1950s, researchers worldwide began to explore sexual function. Unfortunately, most studies focused on male sexual issues (i.e., erectile dysfunction, premature ejaculation)^1, 2^. However, women continue to occupy an increasingly dominant place in the current social and economic life of China. Concurrently, women’s health issues have also become an increasingly mainstream concern, especially within urban areas^3, 4^. Only in recent decades has research begun to shift attention to this topic^5–8^, but the literature regarding Chinese female sexual dysfunction (FSD) is still limited.

FSD is defined as disturbances in sexual desire and psychophysiological changes that characterize the sexual response cycle and causes obvious distress and interpersonal difficulty^9^. According to different studies, the prevalence of FSD ranges from 26.6% to 63% worldwide^10–12^, about 30% in Asia^13^. Thus, FSD is a common difficulty afflicting women worldwide.

FSD is a multifactor-related problem^14, 15^. According to previous studies, psychological (e.g., irritation, depression, anxiety) and socioeconomic factors (e.g., income, educational background, living conditions) lead to FSD^16–18^. Moreover, female sexual function usually is significantly affected by endocrine factors (e.g., prolactin^19^, gonadal hormone^20, 21^). Previously, in a study done on 40 Turkish female patients, Caskurlu concluded that both hypothyroidism and hyperthyroidism are risk factors of FSD. Atis *et al*. reported that the mean total FSFI scores were 24.2 ± 9.96 in the hyperthyroidic group and 29 ± 10.4 in the control group (P < 0.0001) and concluded that hyperthyroidism is a risk factor for FSD^22^. Therefore, thyroid hormone disorder is also believed to be associate with FSD^16^.

In China, the incidence of FSD is poorly understood. The optimal cutoff value was established by Ma^8^ in 2014 based on research conducted within the Chinese Han community, the largest ethnic group in China. Nevertheless, risk factors of FSD in healthy Chinese women remain understudied. Therefore, a preliminary hospital-based screening study investigating risk factors of FSD based on the optimal diagnostic value for Chinese women was conducted in China.

## Materials and Methods

A hospital-based observational study was conducted at the Health Promotion Center of West China Hospital between June and December 2015 based on the center’s prospective database. The center provided routine check-ups for every included woman. Interviewed women who met the following criteria were included: a) had sexual activity in the last 4 weeks and b) completed the Female Sexual Function Index (FSFI) questionnaire satisfactorily. Women who met the following criteria were excluded from enrollment: a) received hormone therapy (e.g., systemic lupus erythematosus, chronic kidney disease) or hormone replacement therapy (e.g., thyroxine replacement), b) was not heterosexual, or c) could not read or understand the content of the questionnaire. After written informed consent was acquired, all participants completed the questionnaire in a separate comfortable room. Volunteers and the physician were available to answer questions during the participants’ self-evaluation.

All participants had a thyroid hormone function test, which evaluated thyroid-stimulating hormone (TSH), free thyroxin (fT4), free triiodothyronine (fT3), thyroid peroxidase antibody (TPOab), and thyroglobulin antibody (Tgab). Participants who had overt hypothyroidism or hyperthyroidism were excluded from the final analysis. The reference intervals for thyroid hormones were as follows: TSH, 0.27–4.2 mU/L; fT3, 3.6–7.5 pmol/L; fT4, 12–22 pmol/L; Tgab, <115 IU/mL; and TPOab, <34 IU/mL. The diagnosis of Hashimoto’s thyroiditis is currently established by a combination of the presence of serum antibodies against thyroid antigens (mainly to thyroperoxidase and thyroglobulin) and appearance on thyroid sonogram^23^.

Female sexual function was evaluated using the Chinese version of the FSFI (CVFSFI), which was validated by Sun *et al*. in 2011^5^. CVFSFI is a well-structured, self-administered screening questionnaire with 19 items regarding female sexual function. The CVFSFI evaluates sexual function or problems during the past 4 weeks in six domains (desire, arousal, lubrication, orgasm, satisfaction, and pain). The raw score on each domain ranges from 1 to 5 or 0 to 5. The total score is obtained by calculating the sum of the six domain scores multiplied by the domain coefficients (0.3–0.6), respectively. In research conducted by Ma *et al*.^8^ in 2014, female sexual dysfunction was defined as a CVFSFI score lower than 23.45. In addition, the optimal cutoff values for low desire, arousal, lubrication, orgasm, satisfaction, and sexual pain in Chinese women were 2.7, 3.15, 4.05, 3.8, 3.6, and 3.8, respectively.

Psychological status was evaluated using the second version of the Beck Depression Inventory (BDI-II), a 21-item self-report inventory that is one of the most widely used psychometric tests and a validated screening tool. This inventory was designed for individuals over 12 years old. Hopelessness, irritability, cognitions and physical symptoms, such as fatigue, weight loss and lack of sexual interest, are the main foci of the BDI-II^24^. Participants scoring 0–13 were classified as having no depression status; 14–19, light depression status; 20–28, medium depression status; and 29–63, severe depression status.

Data analysis was performed using SPSS version 21 (SPSS Inc., Chicago, IL). If the data were normally distributed, continuous variables were presented as the mean ± standard deviation and compared by t-test. If the data were not normally distributed, continuous variables were presented as the median and interquartile range (IQR) and compared by Mann-Whitney U test. Pearson’s chi-square test or Fisher’s exact test was used to compare frequencies (percentages) between categorical variables. Logistic regression was used to identify the risk factors. P value < 0.05 indicated a significant difference.

### Ethics

All experimental protocol in this study involving human participants was approved by the Ethic Committee of West China Hospital, Sichuan University (Chengdu, China). The informed consent forms were obtained from all individual participants included in this study. All study participants provided written informed consent to indicate their agreement for the clinical data to be used in clinical research and publication. The methods were carried out in accordance with the Declaration of Helsinki and the guidelines of the Ethical Committee of the West China Hospital (Chengdu, China).

## Results

After the enrollment period, a total of 1314 women who had sexual activity in the past 4 weeks expressed their interest in participating in the present study. Of these women, 1265 completed the CVFSFI questionnaire satisfactorily. However, 146 participants were excluded on the basis of their thyroid hormone test because of overt hyper- or hypothyroidism. Therefore, 1119 participants with a mean age of 38.7 years were included in the final analysis. No participant was reported as having a disorder of the autonomic nervous system. Thirty-seven participants were diagnosed with non-malignant cervical disease. In general, the mean FSFI score was 25.7 ± 3.9. Based on the optimal cutoff score of 23.45, 297 participants (26.5%) were identified as having FSD. Moreover, the incidence of low desire, low arousal, low lubrication, low orgasm, low satisfaction and sexual pain in participants was 17.2%, 16.6%, 19.8%, 22.0%, 22.8% and 23.6%, respectively.

Specifically, the number of participants aged ≤29, 30–39, 40–49, and ≥50 years was 126, 543, 357 and 105, and the mean FSFI score in each age category was 26.9 ± 3.5, 25.9 ± 3.8, 26.1 ± 3.5, 21.8 ± 3.7, respectively. The incidence of FSD increased from 16.7% to 65.7% as age increased. Similarly, the trend also appeared in other domains (low arousal, lubrication, satisfaction and sexual pain). The basic characteristics of the participants and FSFI score in each domain are shown in Tables 1 and 2.

**Table 1:** Basic characteristics of the participants.

		FSFI < 23.45 (n = 822)	FSFI > 23.45 (n = 297)	P value
Age	38 (33, 43)	37 (33.42)	41 (35, 49.5)	<0.001
BMI	21.7 ± 2.5	21.52 ± 2.42	22.32 ± 2.72	0.012
TSH*	2.46 (1.78, 3.46)	2.36 (1.81, 3.37)	2.69 (1.76, 3.66)	0.484
fT3	5.02 ± 0.71	4.87 ± 0.55	5.45 ± 0.78	<0.001
fT4	17.0 ± 2.2	17.07 ± 2.14	16.72 ± 2.28	0.17
Tgab*	18.96 (14.16, 26.06)	17.91 (13.25, 230.74)	17.8 (14.17, 107.7)	0.335
TPOab*	8.06 (5.21, 13.97)	8.57 (5.48, 14.28)	6.97 (0.00, 12.24)	0.036
Systolic pressure	108.8 ± 13.5	108.37 ± 12.88	109.92 ± 15.20	0.352
Diastolic pressure	68 (63, 74.75)	68 (63, 74)	67 (62, 75.5)	0.962
Hypertension (Yes/No)	63/1056	42/780	21/276	0.239
Diabetes (Yes/No)	42/1077	27/795	15/282	0.211
Hashimoto’s disease (Yes/No)	78/1041	66/756	12/285	0.023
Perimenopause (Yes/No)	228/891	135/687	93/204	<0.001
Menopause (Yes/No)	39/1080	12/810	27/270	<0.001
Smoking				0.245
Never	1104	813	291	
Current	15	9	6	
Alcohol consumption				0.039
Never	1056	783	273	
Rarely	63	39	24	
Marital status				0.531
Married	1089	798	291	
Unmarried	30	24	6	
Educational status				<0.001
≤Middle School	507	339	168	
≥University	612	483	129	
Income (RMB/year)				<0.001
≤40,000	237	123	114	
40,000–100,000	825	666	159	
≥100,000	57	33	24	
Physical activity				0.003
Never	210	135	75	
Rarely	321	240	81	
Frequently	588	447	141	
Depression status				<0.001
No	906	213	693	
Light	180	54	126	
Medium	33	30	3	
Severe	0	0	0	
Parity*	1 (1, 1)	1 (1, 1)	1 (1, 1)	>0.999

**fT3:** free triiodothyronine; **fT4**: free thyroxine; **TPOab**: thyroid peroxidase antibody; **Tgab**: thyroglobulin antibody; **TSH:** thyroid stimulating hormone; **BMI:** body mass index.

*Presented as the median (interquartile range).

P < 0.05 indicates a significant difference.

**Table 2:** The FSFI score and prevalence of FSD in each age category.

Age	General	≤29	30–39	40–49
Characteristics	(n = 1119)	(n = 126)	(n = 540)	(n = 348)
FSFI score	25.7 ± 3.9	26.9 ± 3.5	25.9 ± 3.8	26.1 ± 3.5
Desire	3.4 ± 0.8	3.6 ± 0.9	3.4 ± 0.7	3.5 ± 0.7
Arousal	3.9 ± 0.9	4.1 ± 0.8	3.9 ± 0.8	4.0 ± 0.9
Lubrication	4.9 ± 0.9	5.3 ± 0.7	4.9 ± 0.9	5.0 ± 0.8
Orgasm	4.4 ± 0.8	4.4 ± 0.7	4.4 ± 0.8	4.5 ± 0.8
Satisfaction	4.6 ± 0.8	4.8 ± 0.7	4.6 ± 0.8	4.6 ± 0.8
Sexual Pain	4.5 ± 0.9	4.7 ± 0.8	4.6 ± 0.8	4.5 ± 1.0
FSD (FSFI score < 23.45)	297 (26.5%)	21 (16.7%)	123 (22.8%)	84 (24.1%)
Low Desire (<2.7)	192 (17.2%)	12 (9.5%)	81 (15.0%)	51 (14.7%)
Low Arousal (<3.15)	186 (16.6%)	9 (7.1%)	72 (13.3%)	57 (16.4%)
Low Lubrication (<4.05)	222 (19.8%)	3 (2.4%)	99 (18.3%)	57 (16.4%)
Low Orgasm (<3.8)	246 (22.0%)	27 (21.4%)	105 (19.4%)	69 (19.8%)
Low Satisfaction (<3.6)	255 (22.8%)	21 (4.8%)	117 (21.7%)	78 (22.4%)
Sexual Pain (<3.8)	264 (23.6%)	24 (19.0%)	108 (20.0%)	87 (25.0%)

**FSFI:** Female Sexual Function Index**; FSD:** female sexual dysfunction.

After adjusting for cofounding variables, we found that age (2.115, [1.264, 5.339], P = 0.023), menopause (6.132, [1.520, 24.733], P = 0.011), parity (1.575, [1.075, 2.307], P = 0.020), fT3 level (1.383, [1.011, 1.891], P = 0.043) and depression status (medium risk) (25.659, [2.949, 223.267], P = 0.003) were identified as independent risk factors of FSD, meanwhile income (40,000–100,000 RMB/year) (0.314, [0.170, 0.581], P < 0.001), educational status (≥university) (0.515, [0.294, 0.902], P = 0.020) as protective factors, as shown in Table 3.

**Table 3:** Risk factor identification.

Variables	Univariate analysis	OR	Multivariate analysis	OR	95% CI lower	95% CI upper
	P value		P value			
Age	<0.001	1.068	**0.023**	**2.115**	**1.264**	**5.339**
Income (40,000–100,000 RMB/year)	<0.001	0.258	**<0.001**	**0.262**	**0.131**	**0.521**
Educational status (≥university education)	0.009	0.539	**0.02**	**0.515**	**0.294**	**0.902**
Perimenopause	0.002	2.32	**0.061**	**/**	**/**	**/**
Menopause	0.002	6.75	**0.013**	**5.233**	**1.365**	**22.102**
Marital status	0.059	/	**/**	**/**	**/**	**/**
Physical activity (frequently)	0.173	/	**/**	**/**	**/**	**/**
Parity	0.042	1.384	**0.017**	**1.662**	**1.096**	**2.519**
TSH	0.562	/	**/**	**/**	**/**	**/**
TPOab	0.546	/	**/**	**/**	**/**	**/**
Tgab	0.879	/	**/**	**/**	**/**	**/**
BMI	0.013	1.131	**0.291**	**/**	**/**	**/**
Systolic pressure	0.352	/	**/**	**/**	**/**	**/**
Diastolic pressure	0.734	/	**/**	**/**	**/**	**/**
Hypertension	0.47	/	**/**	**/**	**/**	**/**
Diabetes	0.523	/	**/**	**/**	**/**	**/**
Smoking	0.499	/	**/**	**/**	**/**	**/**
Alcohol consumption	0.222	/	**/**	**/**	**/**	**/**
HD	0.19	/	**/**	**/**	**/**	**/**
fT3	<0.001	3.9	**<0.001**	**4.669**	**2.878**	**7.577**
fT4	0.17	/	**/**	**/**	**/**	**/**
Depression status (medium risk)	0.001	32.535	**0.008**	**19.297**	**2.138**	**174.156**

**fT3:** free triiodothyronine; **fT4**: free thyroxine; **TPOab**: thyroid peroxidase antibody; **Tgab**: thyroglobulin antibody; **TSH:** thyroid stimulating hormone; **BMI:** body mass index.

P < 0.05 indicates a significant difference.

**Figure 1:**
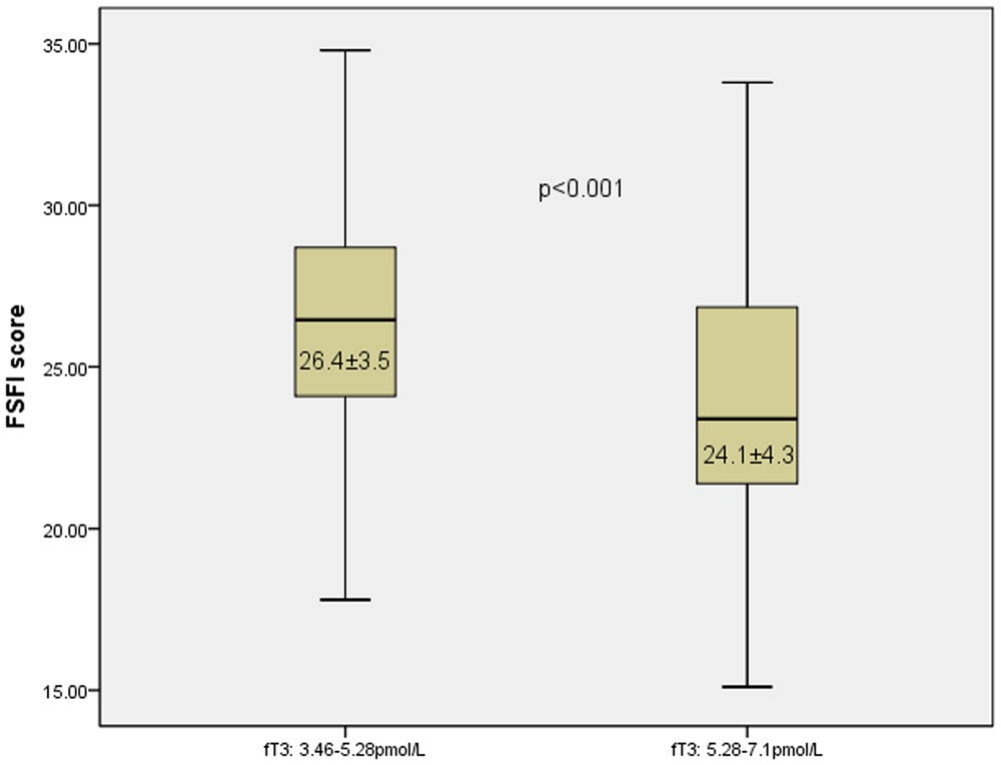
Participants were divided into two groups based on fT3 value (range: 3.46–7.1 pmol/L): 795 (3.46–5.28 pmol/L) and 324 (5.28–7.1 pmol/L). The box-plot indicates that the CVFSFI score differed significantly between the two groups, P < 0.001.

In our study, fT3 level, the values of which ranged from 3.46 to 7.1 pmol/L, was identified as an independent risk factor of FSD. Participants were divided into two groups based on fT3 value: 795 individuals with values ranging from 3.46–5.28 pmol/L and 324 individuals with values ranging from 5.28–7.1 pmol/L. Then, a significant difference was found between the two groups in FSFI score, 26.4 ± 3.5 and 24.1 ± 4.3, respectively, P < 0.001, as shown in Fig. 1.

## Discussion

To our limited knowledge, Italian researcher Pontiroli firstly established the brief relation between thyroid hormone metabolic disorders and erectile dysfunction^25^. Then revealed increased prevalence of female sexual dysfunction (FSD) in hypothyroid subjects related with cardiovascular risk factors^26^. However, sex research is delayed in China in some degrees, the presented study investigating the potential effect of thyroid hormones disorder on FSD among Chinese women is the first one. Although Lee *et al*. first demonstrated that subclinical hypothyroidism is not a risk factor of FSD^27^, they failed to establish the relationship between thyroid hormones and FSD. Our study is also the first hospital-based screening research after Ma *et al*. succeeded in finding that 23.45 was the optimal value for the Chinese population in 2014 on the basis of the Chinese version of the Female Sexual Function Index (CVFSFI) validated in 2011^5, 8^.

Our study suggests that the incidence of FSD in China was exactly 26.5%, less than one-third. Women in our study reported a lower incidence than women in other countries. Laumann *et al*. found that 43% of women were diagnosed with FSD in the United States^28^. In an Iranian population-based study, more than 52% of women had at least one type of FSD^29^. Ibrahim *et al*. reported that 52.8% of interviewed women suffered from FSD in Egypt^30^. In all, the statistical variations were mainly associated with various ethnic backgrounds, different cutoff values and diverse cultural backgrounds. Chinese women always are reluctant to talk about sexual issues openly, which can even prevent them from consulting with physicians due to their traditional conservative beliefs. However, the findings of this study coincided with other studies reporting that the prevalence of FSD in Medellin, Turkey, Malaysia and Iran was 30%, 26.1%, 29.6% and 31.5%, respectively^31–34^.

FSD is a problem that is related to many factors, including psychological, financial, social and physiological factors. The FSFI score and incidence of FSD decreased and increased, respectively, with age. As reported in Simten Malhan’s^35^, Pan Lianjun’s^36^ and Seung Mi Lee’s^27^ studies, age (OR: 2.115, [1.264, 5.339], P = 0.023) was found to be an independent risk factor of FSD in this study. Lower annual income, poor educational background, menopause and depression status were shown to be independent risk factors of FSD in the present study, which was consistent with previous studies^27, 37^. Ahmed *et al*. found that parity had a negative relationship with FSFI score during pregnancy^38^. In contrast, Mostafa *et al*. stated that there was no significant relationship between FSD and gravidity and parity across the three pregnancy trimesters^39^. In this study, however, parity was shown to be a risk factor of FSD in healthy participants. Although the mean of the TPOab in the FSD and non-FSD group was significantly different, however, it was not an independent risk factor in the multivariate analysis. However, Oppo *et al*. reported that thyroid autoimmunity may selectively impair sexual desire independently of thyroid function^40^.

In the present study, elevated fT3 was found to be an independent risk factor of FSD. It is believed that the thyroid hormone level can influence reproductive hormone levels as well as sexual and reproductive function. Hyperthyroidism affects female sexual function through causing an obvious change in steroid metabolism, especially androgen^41^. As we know, androgen is a key factor in the stimulation of female sexual interest and maintenance of desire^42^. Reportedly, hyperthyroidism is associated with a decreased free testosterone concentration and a decrease, in some cases, in estradiol, with a marked increase in sex hormone-binding globulin (SHBG)^20^. Jacobson *et al*. reported that 70% of women complaining of decreased libido had lower testosterone levels^43^. In the current research, although the relationship between hyperthyroidism and FSD was not investigated, elevated fT3 was found to be an independent risk factor of FSD, which was consistent with the known mechanism.

Several limitations were present within our study. First, it was hospital-based research. West China Hospital is located in Chengdu, Sichuan province, southwestern China. Comparatively, the economy is less developed than in the coastal region or Beijing. People tend to be more conservative, especially regarding their sex lives or experiences. Previously, Sun *et al*.^5^ reported that the incidence of FSD was 52% in Beijing, although they used the DSM-IV-TR as a screening tool, not the FSFI. The results in our study are hardly representative of the whole country due to uneven economic and cultural development. As such, we look forward to more research focusing on this topic. Second, to some degree, limited diversity was inevitable because most participants originated from urban locales. The results may have been a little different if the study were conducted in a rural or countryside area. Therefore, a multicenter screening study is needed. Third, due to the limitations of health examination programs, data on sex hormones, such as testosterone, estradiol, and luteinizing hormone, could not be collected. An extensive study involving the FSFI questionnaire and sex hormones is currently in progress. Additionally, it may be interesting to investigate whether thiamazole would affect the FSFI score, quality of life and sex hormone metabolism in hyperthyroidism patients. Moreover, pelvic exams were not performed on participants because of limitations of the check-up program. Reports of incontinence, which has been found to be associated with FSD^44, 45^, were lacking in the study.

In addition, physicians are usually more concerned about TSH and fT4 than fT3 in replacement or suppression therapy in thyroidectomy patients^46^. Maybe fT3 is an unneglectable issue in thyroidectomy patients, especially female patients.

In conclusion, the frequency of female sexual dysfunction in China, specifically the southwestern part of the country, was 26.5%. In addition to annual income, educational background, depression status, menopause and parity, elevated fT3 was closely associated with FSD. In clinical practice, more attention should be given to the relationship between fT3, even overt or subclinical hyperthyroidism, and FSD.
